# Venomics: A Mini-Review

**DOI:** 10.3390/ht7030019

**Published:** 2018-07-23

**Authors:** David Wilson, Norelle L. Daly

**Affiliations:** Centre for Biodiscovery and Molecular, Development of Therapeutics, AITHM, James Cook University, Cairns, QLD 4878, Australia; norelle.daly@jcu.edu.au

**Keywords:** venomics, genomics, transcriptomics, proteomics, pharmacomics, bioinformatics

## Abstract

Venomics is the integration of proteomic, genomic and transcriptomic approaches to study venoms. Advances in these approaches have enabled increasingly more comprehensive analyses of venoms to be carried out, overcoming to some extent the limitations imposed by the complexity of the venoms and the small quantities that are often available. Advances in bioinformatics and high-throughput functional assay screening approaches have also had a significant impact on venomics. A combination of all these techniques is critical for enhancing our knowledge on the complexity of venoms and their potential therapeutic and agricultural applications. Here we highlight recent advances in these fields and their impact on venom analyses.

## 1. Introduction

The complex mixtures of diverse, selective and potent natural products found in the venom of venomous creatures has garnered significant interest and is well-published as a source of potential therapeutic leads [1,2,3,4]. This interest stems from >50% of all approved drugs arising from natural products or their derivatives [5], including six venom-derived drugs approved by the Food and Drug Administration (FDA) [6]. In addition to therapeutic potential, compounds from venoms also have potential as bioinsecticides [7].

**Figure 1:**
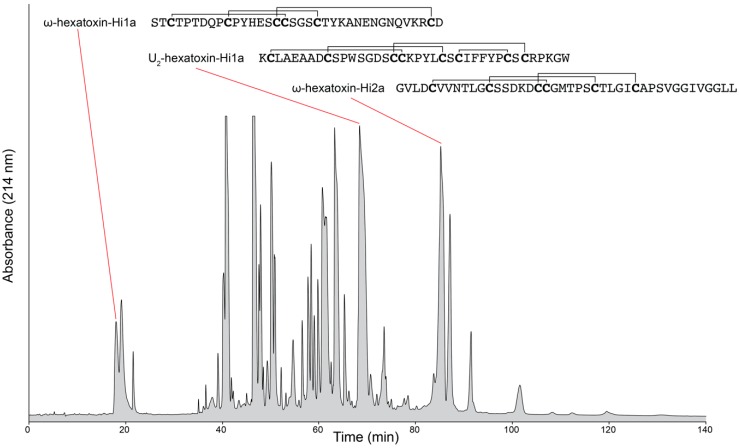
Venom composition of an Australian funnel-web spider. Reversed-phase high-performance liquid chromatography (RP-HPLC) chromatogram of crude venom milked from *Hadronyche infensa*, highlighting the complexity of the venom. The sequences of three known peptides are shown. The sequences are catalogued in Arachnosever [16].

Although the natural chemical libraries contained within venoms are often touted as rich sources of therapeutic and bioinsecticide leads, one of the major challenges facing venom researchers has been the characterization of molecules in highly complex venom mixtures [8]. For instance, individual spider venoms and cone snail venoms are reported to contain upwards of 1000 different components per species [9,10,11]. An example of the complexity of venom composition is shown in the reversed-phase high-performance liquid chromatography chromatogram of crude venom from the Australian funnel-web spider (*Hadronyche infensa)* (Figure 1). Limited quantities of venom have also hindered attempts to characterise venom components [12]. Accessing this expansive peptide reserve requires the broad implementation of multidimensional miniaturised high-throughput strategies [2].

Advances in omics technologies, synonymous with high-throughput techniques [13], such as proteomics, transcriptomics and genomics approaches have facilitated the characterisation of venom peptides and proteins, and led to the term venomics. Venomics was first described as the venom gland proteome [14] but the definition has expanded to encompass the global study of the venom and the venom gland, incorporating characterization of the whole venom profile through integration of proteomic, transcriptomic and genomic methodologies [15]. Without this integration, the individual studies are just a venom-based omics study and lack complementary data.

Classic venomics workflows offer a rapid and relatively inexpensive “solution” to the deconvolution of complex venom compositions at the peptide and protein sequence level. However, the most critical aspect of venoms in the biological sense remains the functional activity. Without biological function data, the acquired information is limited to modelling toxin sequence evolution and structure/activity prediction by homology. Venomics studies need to expand to encompass functional data.

In this review, we discuss the fields and methodologies that combine to form venomics, with a focus on its role in the discovery and identification of novel compounds. We expand on the classic venomics definition and argue the critical requirement of functional biological data, and the inclusion of pharmacomics into the venomics definition. We provide examples from venomics studies, focusing on spiders, and discuss the issues and limitations experienced, and the advances in technology to overcome these hurdles.

## 2. Venomics

### 2.1. Genomics

Knowledge of the full genome can aid venom analysis and biodiscovery where the compounds of interest are primarily direct gene products [17]. The genome contains the coding information for every expressed, and potential, venom peptide and protein. This information is “hidden” in the complex web of genes, exons and introns. 

Currently, genome sequencing is typically performed using third-generation next-generation sequencing (NGS) technologies, having moved forward from the second-generation NGS technologies (e.g., 454 pyrosequencing, sequencing by oligonucleotide ligation and detection (SOLiD), and Illumina reversible terminator chemistry) (reviewed in [2]). The “short-read” sequencing technologies, such as Illumina platforms, have lower error rates and can provide highly accurate genotyping in non-repetitive regions but do not allow contiguous de novo assemblies which restricts the ability to reconstruct repetitive sequences and detect complex structural variation [18]. Longer read lengths are available with single-molecule sequencers (for example, the Pacific Biosciences platform), however these technologies suffer from significantly higher error rates and typically require complementary short read data to assemble high-quality reference genomes de novo [18,19].

Early, selective gene sequencing studies have demonstrated that the genes encoding a number of venom components from the “primitive” mygalomorph spiders *Haplopelma hainanum* [20,21], *Haplopelma huwenum* (Theraphosidae) [22], *Hadronyche infensa* (Atracinae) [23] and a “modern” araneomorph spider *Latrodectus mactans* are intronless [24]. Interestingly, this is in contrast to the araneomorph spider, *Diguetia canities*, where a similar gene structure to cone snail venom peptides is observed [25]. Cone snail genes are structured with short exons (27–226 bp) interspersed with long introns (0.89–1.64 kbp) [26]. However, unlike the cone snails, the *Diguetia canities* spider propeptide and mature toxin are encoded on two separate exons separated by a large intron instead of on single exons [23].

On a broader front, three full genomes have been published from the African social velvet spider, *Stegodyphus mimosarum* (genome size 2.55 Gb), Golden orb-weaver, *Nephila clavipes*, (predicted genome size 3.45 Gb), and common house spider, *Parasteatoda tepidariorum*, (1.5 Gb genome size) and one draft assembly of the Brazilian white-knee tarantula (*Acanthoscurria geniculata*) in genomics studies [17,27,28]. The assembly of the *A. geniculata* genome remains fragmented and a draft due to high heterozygosity, high repeat content and large genome size (estimated to be 6.5 Gb) precluding high quality assembly using short-read Illumina sequencing approaches. An additional two spider genomes, the western black widow (*Latrodectus hesperus*) and brown recluse spider (*Loxosceles reclusa*), are listed in the NCBI Genome database (May 2018).

Advances in sequencing technologies, such as nanopore sequencing, provide longer read and average read lengths and are looking to overcome limitations associated with heterozygosity, high repeat content and large genome size, and improve de novo genome sequencing and assembly [29]. As an example, Loman et al. [30] reported using the Oxford Nanopore Technologies MinION platform to sequence and perform de novo assembly of 133.6 Mb of read data, representing ~29× coverage of the reference genome, into a 4.6 Mb single contig for *Escherichia coli* [30]. Similarly, the same platform was used to sequence the more complex genome of *Saccharomyces cerevisae* and reported upwards of 450 Mb of data per run with an average read length of 5548 bp [31].

More recently, Jain et al. [18] highlighted the challenges faced in assembling complex genomes, such as the human genome, with high accuracy and completeness despite advances in sequencing technology. These challenges stem from the size of the genome (~3.1 Gb), heterozygosity, regions of GC% bias, diverse repeat families, and segmental duplications that contribute to at least 50% of the genome. Pericentromeric, centromeric, and acrocentric short arms of chromosomes, which contain satellite DNA and long tandem repeats, pose even greater challenges. However, in their study they utilised the unamplified DNA on a MinION sequencer to generate 91.2 Gb of sequence data representing ~30× theoretical coverage of a human genome. The nanopore sequence data alone allowed generation of a de novo contiguous assembly with the longest minimum contig length that summed to at least half the haploid genome size (NG50) of 3 Mbp. They developed a protocol to generate ultra-long reads, with read lengths up to 882 kb and minimum read lengths that sum to at least half the bases (N50) of greater than 100 kb. These ultra-long reads provided an additional 5× coverage, for a total of 35×, and doubled the NG50 to 6.4 Mb. The study reported that the read lengths produced were dependent on the input fragment length, and that careful preparation of the DNA samples using classical extraction and purification methods improved read length. Furthermore, they argue there may be no intrinsic read-length limit for pore-based sequencers beyond the physical forces that lead to DNA fragmentation in solution [18]. Long-read sequencing technology still suffers from limitations related to high error rates [32]. However, evidence has shown that an intermediate solution between new advances in sequencing technology to overcome current sequencing limitations may lie in the examination and improvement of protocols, such as sample preparation [18].

Study of the genome combined with transcription expression profiling of tissues can lead to intriguing findings. In studying the genome, and spider silk genes and their expression in the Golden orb-weaving spider (*Nephila clavipes*), Babb et al. [17] discovered an alternatively spliced spidroin (a unique family of structural proteins in spider silk) expressed exclusively in the venom gland [17]. Similarly, Sanggaard et al. [28] used genomics and proteomics in a study showing the presence of a high abundance cysteine-rich secretory protein 3 (CRISP3)-like protein in the Brazilian white-knee tarantula (*Acanthoscurria geniculata*), and three isoforms of a homologous protein in the African social velvet spider (*Stegodyphus mimosarum*) [28]. Cysteine-rich secretory proteins are also reported in the venom of snakes, lizards, and cone snails [33,34]. The protein acts as a serine protease and cleaves the propeptide of the mature venoms peptides in cone snails, and is predicted to have the same function in the tarantula and African social velvet spider [28,33].

### 2.2. Transcriptomics

The transcriptome represents the expression, and the level of expression, of genes within cells and in specific tissues/organs at a specific developmental stage or physiological condition. The primary aims of transcriptomics are: (i) to construct a catalogue of all transcript species, including mRNAs, non-coding RNAs and small RNAs; (ii) to establish the transcriptional structure, in reference to their start sites, 5′ and 3′ ends, splicing patterns and post-transcriptional modifications; to determine the level of antisense transcription occurring in cells; and (iii) to quantify any changing expression levels of transcripts under different conditions and during development [35,36].

Sequencing of the expressed RNA is achieved using similar technologies to DNA sequencing. Consequently, transcriptomics faces similar challenges and limitations experienced in genomics. Current established transcriptomics methods use the extensive throughput of next-generation sequencing-by-synthesis platforms, sequencing complementary DNA (cDNA), and is termed RNA-seq. The cDNA is generated by reverse transcription and commonly primed with either a polydeoxythymine (polyDT) primer, or first fragmenting the RNA and priming with random hexamers [35]. The cDNA is then typically prepared via a method involving PCR into a library for sequencing [37,38]. The incorporation of PCR in the library preparation methods imposes several limitations including bias and reduced complexity compared to the original RNA pool resulting from differing amplification efficiencies that cause reduced or excessive amplification of some RNA species, and loss of any epigenetic information present on the original RNA strand [35,39]. 

Exceptions to the PCR-based library preparations do exist, such as flowcell reverse transcription sequencing (FRT-seq) using Illumina platforms [35,40] and direct single molecule RNA sequencing on the, no longer commercially available, Helicos Biosciences platform [41]. In FRT-seq the first strand cDNA synthesis is performed on single strands of fragmented RNA hybridised to the flowcell surface, which requires relatively large quantities of polyA^+^-selected RNA [35].

Direct single-molecule RNA sequencing is suitable for small sample quantities and uses a stepwise sequencing-by-synthesis approach employing native RNA strands as the sequencing template and direct imaging of incorporated fluorescent nucleotide analogues in massively parallel sequencing [41]. These approaches face limitations arising from a reliance on synthetic copies of the original RNA strand, losing information about modifications, and the short sequence reads generated and associated assembly, which may miss the multiple different isoforms of transcripts that can be formed from alternative splicing processes [42]. Short sequence reads generally cannot span entire transcripts or both sides of splice junctions adequately, missing gene isoforms that can have different transcription start sites, coding sequences and untranslated regions that can produce isoforms with very different functions [43]. Long sequence reads have been reported using a strand-switching library creation protocol coupled with long read sequencing platforms (Pacific Biosciences, and nanopore sequencing) to identify new transcript isoforms in the chicken and *Drosophlia* genomes respectively [44,45].

None of the methods mentioned above directly sequence the source RNA strand and are governed by the processivity and error-rate limitations of reverse transcription [37]. More recently, direct sequencing of the original RNA strand, without amplification, has been successfully demonstrated using Oxford Nanopore Technology nanopore sequence technology. Areas within this technology currently identified for optimization/development include improvement of the basecalling model for higher accuracy and modified base recognition, the isolation of intact transcripts to prevent degraded RNA hindering splice variant detection, refinement of the sequencing process to increase the sequencing speed, and optimization of the software tools for nanopore direct RNA data. One notable approach to improve throughput, potentially through disruption of RNA secondary structure, and provide higher read accuracy in this method is to synthesize a complementary cDNA strand in such a way to create an RNA-cDNA hybrid where the cDNA strand is sequenced immediately following the parent RNA strand. The cDNA strand sequence can be combined with the RNA sequence, which acts as an internal reference, to provide a single, higher accuracy read and de novo identification of modified bases [37].

Numerous examples of the use of transcriptomics approaches for analysis of venoms are emerging and resulting in the discovery of novel peptides. The study mentioned previously (Section 2.1) by Sanggaard et al. [28] combines genomics, transcriptomics and proteomics in an integrated venomics approach for the analysis of venom components in two spiders (Brazilian white-knee tarantula and African social velvet spider). The transcriptomes were sequenced using Illumina Hiseq2000. In addition to information of larger proteins present in the venom, a BLAST against the ArachnoServer database [16] (using criteria of <10 kDa and >5 cysteine residues) indicated the presence of 78 cystine knot-like peptide encoding transcripts in the tarantula transcriptome. Similarly, 28 cystine knot-like peptides were present in the transcriptome of the velvet spider. Many of these peptides were confirmed at the protein level. Cystine knot peptides are extremely widespread in nature, and are particularly prevalent in venoms. A recent transcriptomics and proteomics study on remipede crustaceans employing 454 FLX platform sequence technology indicated that the most highly expressed transcripts for non-enzymatic proteins code for cysteine-rich peptides, including cystine knot peptides [46]. 

In addition to the discovery of cystine knot peptides, transcriptomics approaches have been used for the discovery of cystine-rich peptides with novel disulfide bond architectures. We have recently characterized a conotoxin identified from the transcriptome of *Conus miles* [47]. This peptide, Φ-MiXXVIIA, has a novel cysteine framework, and although the connectivity is the identical to a cystine knot peptide, the topology is different. We showed this peptide had structural similarly to a growth factor protein, granulin. Although structural similarity does not necessarily mean the bioactivity is similar, in this case we showed that MiXXVIIA also promotes cell proliferation consistent with the granulin peptides. This activity would not have been explored had the structural link not been observed [47].

### 2.3. Proteomics

Proteomics was first defined in 1995 as the large-scale characterization of the entire protein complement of a cell line, tissue, or organism. As the definition of proteomics has evolved, diverged and expanded over the years, the goal remains constant; to obtain a global and integrated view of biology through study of all the proteins of a cell at a particular time [48].

Two primary strategies have been employed to study the proteome. The traditional approach, analyses the structure and function of isolated specific proteins using established biochemical and biophysical techniques. Alternatively, the advent of large-scale, systematic measurements of proteomes has allowed the determination of biological insights from proteomic datasets themselves, or in combination with other omics data. Both approaches have been fundamentally transformed by the revolution in powerful mass spectrometry-based instrumentation and protocols with the capability to identify and accurately quantify expressed proteins [49]. For the study of venoms, proteomics provides a comprehensive, multi-faceted approach for the analysis of venom proteins, including sequence, post-translational modifications (PTMs), quantity, regionalisation, and stimulus-dependence of venom protein mobilisation [2]. PTMs are particularly common in venom. For example, cone snail venom peptides are notorious for undergoing a diverse range of PTMs, with up to 75% of amino acids post-translationally modified in individual conopeptides [2,15]. Disulfide bonds and C-terminal amidation are common PTMs found in spider venom peptides, and examples of common PTMs observed in cone snail venom peptides include C-terminal amidation, disulfide bonds, N-terminal pyroglutamylation, proline hydroxylation, valine hydroxylation, tryptophan bromination, γ-carboxylation of glutamic acid, tyrosine sulfation, and *O*-glycosylation [2,15].

Mass spectrometry is particularly attractive to proteomics studies, in principle, owing to its inherent specificity of identification, generic proteomic workflow protocols and potential extreme sensitivity [49]. These reasons are especially relevant to venom proteomic studies where sample availability is typically extremely limited and identification of low abundance components within the highly complex mixtures is required. In practice, reaching the full potential of the technique has been challenging and has not been realised [49].

Proteomic studies are currently conducted via two possible approaches; bottom-up proteomics and top-down proteomics. Top-down proteomics is more attractive in theory as it studies the proteins as intact entities, and has the advantage of simultaneously measuring all modifications that occur on the same molecule and enabling identification of the precise proteoform. However, because each protein may have multiple proteoforms (toxiforms in venom [50]) that may have different functions, top-down proteomics is experimentally and computationally more challenging [49]. Additionally, top-down proteomics still faces challenges imposed by current limitations on the front-end fractionation of complex mixtures and instrument-related limitations, particularly in relation to high mass proteins [51].

Bottom-up proteomics has been more experimentally and computationally feasible and is currently the most common approach. In bottom-up proteomics, small peptides are generated by enzymatic digestion of the source protein mixture. The resulting peptide mixture is separated using reversed-phase high performance liquid chromatography and transferred directly to an online mass spectrometer. The peptides are then fragmented in one of three main approaches: data-dependent acquisition (DDA), directed at obtaining complete and unbiased coverage of the proteome; selected reaction monitoring for reproducible, sensitive and streamlined acquisition of particular peptides of interest; and data-independent acquisition to obtain a comprehensive fragment-ion map of the sample. Each approach has advantages and limitations, and hybrid methods are predicted to emerge in the future [49]. The acquired data is then interrogated over a relevant database for protein identification.

The availability of relevant protein or nucleic acid databases is frequently a limitation in proteomic studies [15]. In general, the traditional bottom-up approaches have the disadvantage of typically failing to provide complete protein sequence coverage and preventing the distinction between different related protein species, particularly proteoforms and protein isoforms, and is known as the protein inference problem [50,52].

As an example, Sanggaard et al. [28] used bottom-up proteomics together with an assembled genome and venom gland transcriptome of the African social velvet spider (*Stegodyphus mimosarum*), and a fragmented genome and venom gland transcriptome of the Brazilian white-knee tarantula (*Acanthoscurria geniculata*), to build proteomes of the two spiders [28]. A total of 157 venom proteins were identified for the African social velvet spider and 120 venom proteins for the Brazilian white-knee tarantula. These results are significantly lower than the previously reported >600 masses detected in the venom of female *Atrax robustus*, or 1000 masses detected in female *Hadronyche versuta* venom [53]. Similarly, in a study of the transcriptome and proteome of the mygalomorph Brush-foot trapdoor spider (*Trittame loki*), a total of 46 venom proteins were identified and their presence in the venom confirmed, with the exception of the Kunitz protein, by proteomics [54]. This discrepancy could be the result of unoptimized methods for the proteomes, or simply less complex venom compositions [53].

The critical requirement for the mass spectrometry data of proteomics to feed into bioinformatics analysis studies (see Section 2.4.) for protein identification and quantitation, necessitates the integration of proteomics into the venomics workflow [2]. As advances and developments in technology overcome the fractionation, instrumentation and software hurdles currently faced, top-down proteomics will rise to the fore with its promise to provide a global and integrated inventory of all the proteins of a cell at a particular time.

A subset of proteomics studies that has previously been incorporated into venomics studies is glycomics. The identification of glycosylated peptides and proteins in venom can be highly important in venom-based therapeutic lead discovery, particularly the discrimination between carbohydrate- and protein-based epitopes as the source of an allergic response. High-resolution mass spectrometers, such as the combined ion trap and triple quadrupole Q-Trap instruments, can be used to identify intact glycoproteins and characterize glycans following chemical or enzymatic cleavage and derivatization [55,56,57].

### 2.4. Bioinformatics

Bioinformatics integrates the data obtained from the genomic, transcriptomic and proteomic studies to provide a more complete picture of the venome. A number of databases are available as a resource for venomics studies. The NCBI and Uniprot’s animal toxin annotation project databases offer a general resource of animal toxins, while a number of specifically focused databases are available and include potassium channel toxins (Kalium [58]), spiders (Arachnoserver [16]), cone snails (ConoServer [59]) and snakes of Bangladesh (ISOB [60]). As an example, Arachnoserver [16] acts as a specialised repository database of known and newly discovered spider venom peptides and proteins. The database can be used as a reference in the annotation of spider genomic and transcriptomic data, and as a reference for proteomic studies.

Arachnoserver also provides a spider toxin annotation and evaluation facility in Tox|Note, a bioinformatic pipeline designed to fast-track the analysis of spider venom-gland transcriptome data generated by next-generation sequencing, and allows annotation of toxin transcripts, prediction of signal and propeptide cleavage sites in full-length toxin precursor sequences, and automatic generation of rational toxin names based on the published nomenclature rules [61]. As an example, transcriptome data was obtained from next-generation sequencing of venom gland RNA isolated from the dissected venom glands of a species of Australian theraphosid (*Phlogius* sp.). The extracted RNA sample was sequenced using Illumina HiSeq 2000 technology at the Ramaciotti Centre for Genomics and provided 40.14 Gb of sequence data. The data was assembled de novo using the Trinity software (v2.2.0) to generate a total of 141,365 contigs and 84,809 gene clusters. Annotation by submission of the contig data file to Tox|Blast yielded 121 spider toxin open reading frames [62]. Similarly, ConoSorter, from The University of Queensland in Australia, is a high-throughput standalone tool for large-scale identification and classification of precursor conopeptides into gene superfamilies and classes from raw NGS transcriptomic or proteomic data [63].

### 2.5. High-Throughtput Assay Screening

The missing link between classical venomics and relevant therapeutic lead identification is the use of high-throughput biochemical and functional assay technologies to screen expansive compound libraries. Improvements in technology have significantly increased the capacity and automation of high-throughput screens, while simultaneously reducing the amount of sample required. The assay technologies available include traditional assays like electrophysiology, absorbance/fluorescence-based assays, radioligand binding, and enzyme-linked immunosorbent assays (ELISAs). More recently developed technologies include AlphaScreen and label-free technologies such as XCELLigence, and bioluminescence, fluorescence, polarization, fluorescence-resonance energy transfer (FRET), bioluminescence resonance energy transfer, and scintillation proximity assays. The advantages and limitations of these technologies have been reviewed by Vetter et al. [64,65].

These high-throughput technologies are generally useful for the range of biological targets venom components act upon, often with exquisite selectivity and potency, including ion channels, G-protein coupled receptors, transporters and enzymes [65]. The basic requirements of high-throughput screens include high sensitivity and accuracy, and robustness and reproducibility, which can be evaluated using statistical tools such as the Z-factor [66]. However, in contrast to screening combinatorial chemical libraries, assay of venoms composed of mixtures of molecules with diverse biological effects can suffer from interference from non-target-specific interactions. While the traditional approach of increased miniaturization and automation to increase assay capacity is still valid, it can be argued that, in the context of venomics, greater emphasis on data quality is required [64].

**Figure 2:**
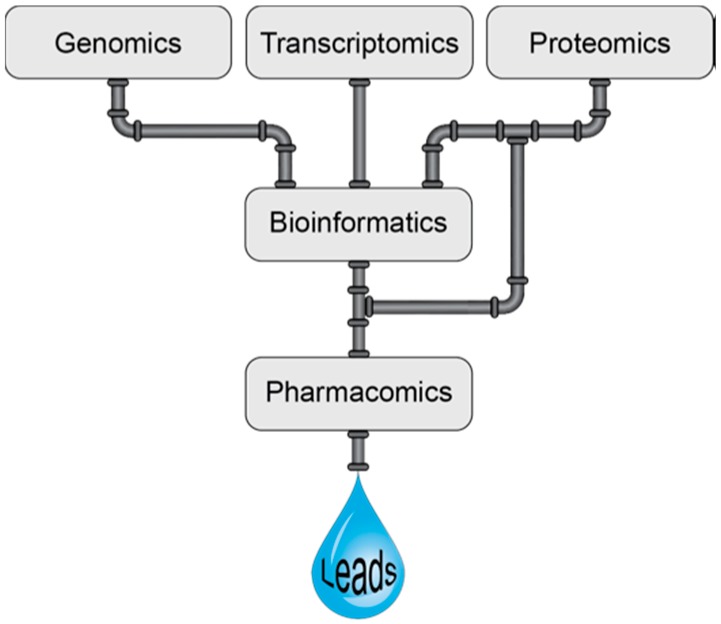
Schematic representation of the venomics pipeline in venom based therapeutic lead discovery.

Many of the high-throughput screening approaches applied to venoms involve pharmacology screening and can perhaps be considered as a branch of pharmacomics, a term coined by Milward et al. [67]. Pharmacomics has been defined as the integration of “omics” approaches to study dynamic molecular states, for monitoring disease states and drug responses [67]. Here we propose the definition be expanded to include the high-throughput pharmacological analysis of venom components. The combination of pharmacomics and venomics is likely to be a powerful alliance for the development of novel drugs as highlighted in Figure 2. However, it should be noted that in the early stages high-throughput screening encompassing biochemical approaches, among others, is also likely to lead to useful information that can ultimately be used in the drug design process. Furthermore, although venom components are generally highly selective, they can have off-targets effects and it will be increasingly more important to determine the primary target for these components to facilitate drug design applications. Computational methods have the potential to aid in this development as reviewed by Kuyucak and Norton [68].

Recent examples of the use of high-throughput assay screening on venoms include the analysis of the venom from the wasp *Nasonia vitripennis*, which has indicated that it might have therapeutic potential. The use of reporter arrays showed the venom altered the expression of nuclear factor κβ (NF-κB) signalling pathway genes that have a role in inflammatory diseases and cancer [69]. Furthermore, a combined cytotoxicity screening and venom profiling approach on snake venom revealed the presence of activity against a human lung carcinoma cell line, and identification of a range of proteins including phospholipases and serine proteases [70].

## 3. Conclusions

The integration of all the technologies discussed above enable a “rapid” deconvolution of the complex mixtures present in venoms, and in combination with high-throughput screening approaches can help to identify new drug leads. The combination of proteomics approaches in addition to genomic and transcriptomic approaches is particularly important for venom studies given the high propensity and diversity of post-translational modifications that can be present.

It should be noted that the focus of these approaches on the proteome excludes the small molecules often present in venoms. We have recently used a cell-based screening approach to discover an acylpolyamine, PA_366_, from an Australian theraphosid species (*Phlogius* sp.) with selectivity toxicity against breast cancer cells [1]. As this small molecule is not a direct gene product, it was not able to be identified in the study of the transcriptome of this species (see Section 2.4). This example serves to highlight the inclusive nature of high-throughput screening approaches when applied to venoms, which are not just restricted to the analysis of the proteome but rather include all the molecular components. Furthermore, it highlights a limitation of automated workflow processes employed in venomics studies and a need to consider a range of additional techniques for venom characterization. For instance, the incorporation of techniques such as liquid chromatography–nuclear magnetic resonance (LC–NMR) might assist in the identification of small molecules in venom and provide a more comprehensive view of the molecular diversity present in venomes.
